# Serum adropin levels are reduced in patients with inflammatory bowel diseases

**DOI:** 10.1038/s41598-020-66254-9

**Published:** 2020-06-09

**Authors:** Darko Brnić, Dinko Martinovic, Piero Marin Zivkovic, Daria Tokic, Ivana Tadin Hadjina, Doris Rusic, Marino Vilovic, Daniela Supe-Domic, Ante Tonkic, Josko Bozic

**Affiliations:** 1 Department of Gastroenterology, University Hospital of Split, Split, Croatia; 2 Institute of Emergency Medicine of Split-Dalmatia County, Split, Croatia; 3 Department of Pathophysiology, University of Split School of Medicine, Split, Croatia; 4 Department of Pharmacy, University of Split School of Medicine, Split, Croatia; 5 Department of Medical Laboratory Diagnostics, University Hospital of Split, Split, Croatia; 6 Department of Health Studies, University of Split, Split, Croatia; 7 Department of Internal Medicine, University of Split School of Medicine, Split, Croatia

**Keywords:** Biomarkers, Inflammatory bowel disease

## Abstract

Adropin is a novel peptide mostly associated with energy homeostasis and vascular protection. To our knowledge, there are no studies that investigated its relationship with inflammatory bowel diseases (IBD). The aim of this study was to compare serum adropin levels between 55 patients with IBD (30 Ulcerative colitis (UC) patients, 25 Crohn’s disease (CD) patients) and 50 age/gender matched controls. Furthermore, we explored adropin correlations with IBD severity scores, hsCRP, fecal calprotectin, fasting glucose and insulin levels. Serum adropin levels were significantly lower in patients with IBD in comparison with the control group (2.89 ± 0.94 vs 3.37 ± 0.60 ng/mL, *P *= 0.002), while there was no significant difference in comparison of UC patients with CD patients (P = 0.585). Furthermore, there was a negative correlation between adropin and fecal calprotectin (r = −0.303, *P *= 0.025), whereas in the total study population, we found a significant negative correlation with fasting glucose levels (r = −0.222, *P *= 0.023). A multivariable logistic regression showed that serum adropin was a significant predictor of positive IBD status when enumerated along with baseline characteristics (OR 0.455, 95% CI 0.251–0.823, *P *= 0.009). Our findings imply that adropin could be involved in complex pathophysiology of IBD, but further larger scale studies are needed to address these findings.

## Introduction

Inflammatory bowel disease (IBD) is a chronic relapsing inflammatory disorder of unknown etiology and unpredictable course. It is mainly classified into two disorders: ulcerative colitis (UC) and Crohn’s disease (CD). They both have a similar clinical manifestation whereas they differ in the location and depth of the inflammation. They are also associated with extraintestinal manifestations which can affect the skin, joints, eyes, liver and neurocognitive performance^1,2^. Global prevalence of IBD is on the rise, as the number of countries adopting “western lifestyle” increases^3^. Although the exact etiology of IBD is unknown, it is currently considered to be multifactorial and that is fabricated through complex interactions between genetic predisposition, environmental factors, immune disorders and microbial disturbances^4^.

Special interest of this study is adropin, a novel peptide which acts as an energy regulator through lipid and glucose metabolism^5^. It is encoded by Energy Homeostasis Associated Gene (*ENHO)* expressed in the liver and brain, but its presence is also proven in muscle, heart, pancreas, and kidneys^6^. A study performed on mice pointed to the role of adropin as a physiological regulator of glucose metabolism and oxidation of fatty acids. It was found that mice with diet induced obesity, when treated with adropin, showed increased glucose tolerance, reduced insulin resistance, and the promotion of carbohydrates in oxidative reactions^7^. A study conducted on patients presented the first evidence pointing to the link between adropin, obesity and risk of metabolic syndrome. It was found that the concentration of adropin were decreased in subjects with obesity and insulin resistance, and that loss of body weight led to an increase in adropin levels^8^. Regarding cardiovascular effects, several recent studies linked low adropin levels with high blood pressure. Negative correlation of adropin with arterial blood pressure, as with the levels of vasoconstriction factor endothelin-1, speaks in favor of the beneficial effect of adropin as a vascular protector^9,10^. Moreover, adropin is involved in neovascularization and vascular protection through the promotion of endothelial nitric oxide (NO) by regulating vascular endothelial growth factor receptor – 2 (VEGF2) and endothelial nitric oxide synthase (eNOS) pathways^11^.

It is indicative by several studies that patients with IBD develop hyperglycemia, insulin resistance and they also have a higher prevalence of diabetes mellitus compared to the general population^12–15^. Inflammation is considered to play a major role in these metabolic disorders and some studies are pointing that they are conditioned by the active phase of IBD^16^. TNF-α is lately regarded as one of the main inflammatory drivers in the exacerbation of IBD^17^, and amid its numerous proinflammation effects, TNF-α down regulates eNOS which consequently leads to reduced perfusion, higher leukocyte infiltration, impaired wound healing and endothelial dysfunction^18^. Given that it was reported by several studies conducted on chronic inflammatory diseases that adropin has a significant correlation with TNF-α^19–21^ and since a study conducted on nonhuman primates reported expression of *ENHO* gene in terminal ileum and colon^22^, its worthy exploring could adropin be somehow involved in complex IBD pathophysiology. The possible metabolic, immunomodulatory and protective role of adropin stresses the importance of further necessary research in this field.

With extensive analysis of available literature, there is no published study that investigated adropin association with IBD. Hence, the aim of this study was to determine serum adropin levels in patients with IBD in comparison with healthy controls. Additionally, we wanted to evaluate the relationship between adropin and UC and CD severity scores, glucose metabolism parameters and inflammatory biomarkers.

## Methods

### Study design

This cross-sectional study was performed at the University Hospital of Split and the University of Split School of Medicine during the period from 1 December 2017 to 1 June 2018.

### Ethical considerations

All subjects were informed about the procedures, course and purpose of the study in a timely manner. Before the start of the study every participant individually signed an informed written consent. The study was approved by the Ethics Committee of University Hospital of Split (date of approval: 23/11/2017) and University of Split School of Medicine (date of approval: 27/11/2017), and was conducted in accordance with all ethical principles of the Seventh Revision of the Helsinki Declaration from 2013.

### Subjects

This study included 55 adult patients with pre-diagnosed IBD (30 patients with UC and 25 patients with CD) and 50 healthy controls. Diagnosis of UC and CD is based on medical history and clinical, radiological, endoscopic and histological features in accordance with the European Consensus on Crohn’s Disease and Ulcerative Colitis (ECCO)^23^. The control group consisted of healthy volunteers, matched with the age and gender of the investigated group. Inclusion criteria were: disease duration of at least one year, stable disease activity in the past 3 months and age between 18 and 65 years. Stable disease activity is referring to clinical evaluation of the disease activity. Using the medical database, we checked did any of the patients contacted a physician regarding new disturbances, worsening and/or had a need for therapy revision. Exclusion criteria were: diabetes; cardiovascular disorders; therapy with corticosteroids during 3 months prior to study onset; substance abuse and consumption of alcohol more than 40 grams/day. We checked medical records of the control subjects regarding gastrointestinal conditions and additionally we performed screening for abdominal pain presence, symptoms related to defecation, change in frequency and form of stool according to the Rome IV criteria for irritable bowel syndrome^24^, as well as any other symptoms which could suggest gluten and lactose intolerance. If any of these conditions were present, we excluded the subject from the control group. Furthermore, all potential control group subjects undergone detailed physical examination along with laboratory analysis of the complete blood count, differential blood count and levels of high sensitivity C-reactive protein. We excluded all participants who showed any sign of inflammation in any of these steps.

### Disease severity assessment

Disease activity was evaluated using clinical and endoscopic indices. Assessment was performed by the same experienced gastroenterologist according to the latest guidelines and the colonoscopy needed for the evaluation of the disease activity was performed within 2 weeks of blood sampling.

Ulcerative colitis endoscopic index of severity (UCEIS) is a quantitative score for grading mucosal inflammation based on the specific endoscopic findings. Three parameters are graded: vascular pattern; bleeding; erosions and ulcers. Depending on the score there are four possible grades for disease activity: remission (0–1); mild (2–4); moderate (5–6); and severe (7–8)^25^.

Mayo endoscopic score (MES) is an instrument used for evaluation of UC through endoscopic findings. There are four possible stages: 0 - remission; 1 - mild disease with erythema, mild friability and decreased vascular patterns; 2 - moderate disease with marked erythema, friability, erosions and absence of vascular patterns; 3 - severe disease with spontaneous bleeding and ulcerations^26^.

Mayo score is a score used for evaluation of UC activity based on four categories (bleeding, stool frequency, physician assessment, and endoscopic appearance). The results correlate with disease severity: <2 remission; 3–5 mild; 6–10 moderate; 10–12 severe^27^.

Simple endoscopic score for Crohn’s disease (SES-CD) is a grading system used for endoscopic evaluation of CD activity. According to the majority of studies the threshold values for interpretation of the results are: ≤2 remission; 2–7 mild activity; 7–16 moderate activity; and ≥16 severe disease^28^.

Crohn’s disease activity index (CDAI) is a quantitative score for the assessment of CD activity based on clinically reported signs, laboratory results and patient reported symptoms in a 7 day period. The three possible grades depending on the result are: clinical remission <150; mild to moderate activity 150–450; and severe disease >450^29^.

Harvey-Bradshaw index (HBI) is a simplified version of the CDAI for assessment of CD activity. Threshold values for the four possible grades are: clinical remission <5; mild activity 5–7; moderate activity 8–16; and severe disease>16^30^.

According to the latest ECCO guidelines, clinical indices should be cautiously used since they are not well-validated in the clinical practice and inconsistencies could be seen^31^. Hence, patients were classified using only the endoscopic scores, while clinical index scores were only descriptively reported. UC disease activity was assessed with two endoscopic index scores (UCEIS; MES) and CD disease activity evaluation was performed with one endoscopic score (SES-CD). In the case of discordance between UCEIS and MES score, results of UCEIS were followed due to its methodological superiority and validity which is in accordance to the latest ECCO guidelines.

### Blood sampling and laboratory analysis

Blood samples were taken after 12-hour fasting from the cubital vein in test tubes with anticoagulant (K3-EDTA) via polyethylene catheter, and after extraction were centrifuged and stored at −80 °C for further analysis. Serum concentrations of adropin were determined using the dual enzyme-linked immunosorbent assay (ELISA) of human adropin (Phoenix Pharmaceuticals, Burlingame, CA, USA), according to the manufacturer’s instructions. The assay detects immunoreactivity of adropin (34–76) in human, rat and mice serum, and there is a 100% homology between peptides. Calibrations were double measured, optical density (OD) values were in accordance with predefined OD values stated in manufacturer instructions and coefficient of variability (CV) of paired calibrations were <15%. Concentration of the analyzed quality control sample which arrived from the manufacturer was within predefined acceptable deviation. The linear range of the assay was 0.3–8.2 ng/mL and sensitivity was 0.3 ng/mL. CV within the probe was less than 10%, and between probes was less than 15%. All blood samples were processed according to international standards, in the same laboratory, by the same, experienced medical biochemist. Moreover, biochemist was blinded to the subjects group in the study. Other biochemical parameters were analyzed according to standard laboratory procedures.

Stool samples were received by a trained laboratory technician in sterile containers within 3 days of sampling. Fecal extraction and dilution to the final concentration of 1:500 was performed with Buhlmann Calex Cap device (Buhlmann Laboratories AG, Schonenbuch, Switzerland) according to the manufacturer instructions. Fecal calprotectin (FC) concentrations were measured by turbidimetric immunoassay method using Buhlmann fCAL turbo assay (Buhlmann Laboratories AG, Schonenbuch, Switzerland) and quantification was performed with the biochemical analyzer Beckman Coulter AU680 (Beckman Coulter, Brea, CA, USA).

### Anthropometric measurements and clinical examination

All participants were subjected to detailed anamnesis, physical examination and measurements of anthropometric characteristics - body weight, body height, body mass index (BMI), waist circumference and hip circumference. For measurement of body weight and height, a calibrated medical scale with built-in heights (Seca, Birmingham, UK) was used. BMI was calculated according to the formula = [body weight (kg)] / [height per square (m2)]. The waist circumference was measured in the middle distance between the bottom of the rib cage in middle axillary line and the tip of the iliac crest in the standing upright position of the examinee. The circumference of the hips was measured at the level of the largest circumference of the gluteal muscles, above the line connecting the large trochanters of the femur.

### Statistical analysis

Sample size analysis has been conducted using data from a pilot study on 10 randomly selected subjects from the patient population (5 patients with UC and 5 patients with CD). Also, data from 10 randomly selected matched control patients was obtained. The value of serum adropin levels, which was the main outcome of the study, was used for the calculation. In IBD patients, the mean adropin levels were 2.99 ± 0.95 ng/mL, and 3.58 ± 0.78 ng/mL in the control group. With type I error of 0.05, and the power of 90%, the required sample size was 47 participants per group.

Collected data was analyzed with statistical software MedCalc (MedCalc Software, Ostend, Belgium, version 17.4.1). Quantitative data was expressed as mean ± standard deviation or median and interquartile range, while qualitative data was expressed as whole number and percentage. Kolmogorov-Smirnov test was used to estimate the normality of data distribution. Comparison of serum adropin levels and other parameters between patients with IBD and control subjects was done by Student t-test for independent samples. For comparison of qualitative variables, Chi-squared test was used. Pearson’s correlation or Spearman’s correlation was used to estimate the correlation between biochemical, anthropometric and clinical parameters with serum adropin levels. Additionally, multivariable logistic regression analysis of independent predictors for positive IBD status was performed, with reporting OR, 95% CI and p-value. The level of statistical significance is set at *P* < 0.05.

## Results

### Baseline characteristics and laboratory parameters

There were no statistically significant differences in age, gender and anthropometric features between the IBD patients and the control group (*P* > 0.05; for all analysis) (Table 1). According to the endoscopic scores 4 patients were in remission, 6 had mild disease, 35 moderate and 10 severe form of disease. UC patients had moderate to severe endoscopic form of disease, while majority of CD patients had moderate endoscopic disease activity. Laboratory analysis showed significantly lower hemoglobin (137.5 ± 21.1 vs 148.4 ± 12.9 g/L, *P* = 0.001), urea (4.4 ± 1.3 vs 5.4 ± 1.5 mmol/L, *P* = < 0.001) and albumin levels (39.5 ± 5.6 vs 44.1 ± 2.6 g/L, *P* = < 0.001) in IBD patients compared to the control group (Table 2).

**Table 1:** Baseline characteristics of IBD patients and healthy controls.

Parameter	IBD group (*N* = 55)	Control group (*N* = 50)	P*
Male gender (*N*, %)	32 (58.2)	30 (60.0)	0.850
Age (years)	39.2 ± 14.2	37.1 ± 12.6	0.421
Body weight (kg)	73.9 ± 15.1	78.7 ± 12.5	0.080
Body height (cm)	176.5 ± 10.1	179.7 ± 9.2	0.101
Body mass index (kg/m^2^)	23.6 ± 3.9	24.3 ± 2.5	0.292
Waist circumference (cm)	86.1 ± 13.4	86.3 ± 11.1	0.935
Hip circumference (cm)	95.1 ± 15.2	98.5 ± 12.8	0.205
Disease duration (years)	8.4 ± 7.9	—	—
IBD-associated hospitalizations	2.0 (0.0–3.0)	—	—
Extraintestinal manifestations (*N*, %)	19 (34.5)	—	—
UCEIS score^†^	6.0 (5.0–7.0)	—	—
MES score^†^	2.5 (2.0–3.0)	—	—
Mayo score^†^	4.5 (2.0–7.0)	—	—
SES-CD^‡^	8.0 (3–12)	—	—
HBI score^‡^	4.0 (1.7–6.0)	—	—
CDAI score^‡^	56.0 (29.0–133.0)	—	—
Aminosalycilates	36 (65.4%)	—	—
DMARD	19 (34.5%)	—	—
Monoclonal Antibodies	33 (60.0%)	—	—

Abbreviations: UCEIS - Ulcerative Colitis Endoscopic Index of Severity; MES - Mayo Endoscopic Score; SES-CD - Simple Endoscopic Score for Crohn’s Disease; HBI - Harvey Bradshaw Index; CDAI - Crohn’s Disease Activity Index; DMARD - Disease-modifying antirheumatic drug.

Data are presented as mean ± standard deviation or median (IQR).

*chi-square test or t-test for independent samples.

^†^Ulcerative colitis group (*N* = 30).

^‡^Crohn’s disease group (*N* = 25).

**Table 2:** Laboratory parameters of IBD patients and healthy controls.

Parameter	IBD group (*N* = 55)	Control group (*N* = 50)	P*
Hemoglobin (g/L)	137.5 ± 21.1	148.4 ± 12.9	0.001
Fasting glucose (mmol/L)	5.0 ± 0.8	5.1 ± 0.7	0.594
Fasting insulin (pmol/L)	61.5 ± 16.5	68.1 ± 19.0	0.117
Urea (mmol/L)	4.4 ± 1.3	5.4 ± 1.5	<0.001
Uric acid (μmol/L)	276.5 ± 76.4	297.3 ± 77.2	0.169
Total bilirubin (μmol/L)	12.9 ± 8.0	15.6 ± 8.7	0.097
Total proteins (g/L)	72.6 ± 7.6	73.2 ± 3.7	0.581
Albumins (g/L)	39.5 ± 5.6	44.1 ± 2.6	<0.001
hsCRP (mg/L)	6.4 ± 3.01	1.37 ± 0.95	<0.001
Triglycerides (mmol/L)	1.33 ± 1.26	1.24 ± 0.6	0.672
Total cholesterol (mmol/L)	5.02 ± 1.52	5.25 ± 1.19	0.391
HDL cholesterol (mmol/L)	1.37 ± 0.43	1.41 ± 0.32	0.630
LDL cholesterol (mmol/L)	2.98 ± 1.2	3.25 ± 1.1	0.237
Fecal calprotectin (μg/g)	220 (13.5–632)	—	—

Abbreviations: hsCRP- high sensitivity C-reactive protein.

Data are presented as mean ± standard deviation or median (IQR).

*t-test for independent samples.

### Serum adropin levels in patients with IBD and control subjects

**Figure 1:**
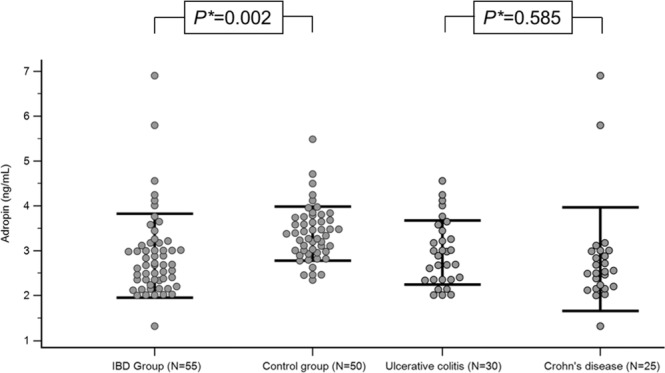
Serum adropin levels in IBD group and control group (**A**) and in ulcerative colitis group and Crohn’s disease group (**B**). *t-test for independent samples.

Serum adropin levels were significantly lower in patients with IBD in comparison with the control group (2.89 ± 0.94 vs 3.37 ± 0.60 ng/mL, *P* = 0.002) and there was no significant difference in comparison of UC patients with CD patients (2.96 ±0.71 vs 2.81 ±1.16 ng/mL, *P* = 0.585) (Fig. 1).

### Correlation between adropin and other parameters

**Figure 2:**
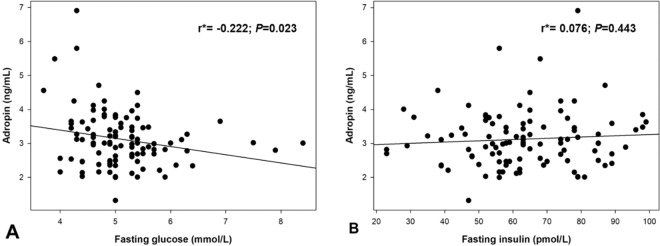
Correlation analysis of adropin levels with fasting glucose (**A**) and insulin levels (**B**) in total study population (*N* = 105). *Pearson’s correlation coefficient.

In the IBD group serum adropin levels weren’t in a significant correlation with serum levels of hsCRP (r = −0.101, *P* = 0.461) whereas in the total study population there was a significant negative correlation (r = −0.235, *P* = 0.015) (Table 3). Additionally, in the IBD group a significant negative correlation was established between serum adropin levels and fecal calprotectin levels (r = −0.303, *P* = 0.025), whereas in the total study population, a significant negative correlation with fasting glucose levels was found (r = −0.222, *P* = 0.023) (Table 3) (Fig. 2).

**Table 3:** Correlation analysis between serum adropin levels and different biochemical and anthropometric parameters.

Parameter	Adropin (ng/mL)	
	Total population (*N* = 105) r^*^(*P*)	IBD group (*N* = 55) r^*^(*P*)
Total proteins (g/L)	−0.075 (0.449)	−0.135 (0.326)
Albumins (g/L)	0.124 (0.208)	−0.074 (0.589)
Triglycerides (mmol/L)	−0.184 (0.059)	−0.103 (0.456)
Total cholesterol (mmol/L)	−0.031 (0.754)	−0.084 (0.542)
HDL cholesterol (mmol/L)	0.123 (0.211)	−0.036 (0.797)
LDL cholesterol (mmol/L)	−0.008 (0.934)	−0.055 (0.692)
Age (years)	−0.268 (0.087)	−0.069 (0.614)
Body mass index (kg/m^2^)	0.040 (0.688)	0.029 (0.834)
Waist circumference (cm)	−0.047 (0.636)	−0.004 (0.975)
Fecal calprotectin (µg/g)	—	−0.303 (0.025)
hsCRP (mg/l)	−0.235 (0.015)	−0.101 (0.461)

Abbreviations: hsCRP- high sensitivity C-reactive protein; IBD- Inflammatory Bowel Disease.

^*^Pearson’s correlation coefficient.

Moreover, multiple linear regression analysis showed that adropin levels retained significant association with fecal calprotectin (β ± SE, 0.003 ± 0.001, *P* = 0.014) after model adjustment for age, BMI and gender, with serum adropin levels as dependent variable.

Regarding the IBD severity scores, in UC patients we found a significant negative correlation between serum adropin levels and UCEIS (r = −0.372, *P* = 0.043), and Mayo (r = −0.369, *P* = 0.044) scores, whereas in CD patients we found a significant negative correlation between serum adropin levels and SES-CD (r = −0.514, *P* = 0.008), HBI (r = − 0.568, *P* = 0.003), and CDAI (r = −0.469, *P* = 0.018) scores (Table 4).

**Table 4:** Correlation analysis between different IBD severity scores and serum adropin levels.

Parameter	Adropin (ng/mL)	
	r^*^	*P*
***Ulcerative colitis (N* = *30)***		
UCEIS score	−0.372	0.043
Mayo score	−0.369	0.044
***Crohn’s disease (N* = *25)***		
SES-CD	−0.514	0.008
HBI score	−0.568	0.003
CDAI score	−0.469	0.018

Abbreviations: UCEIS- Ulcerative Colitis Endoscopic Index of Severity; SES-CD - Simple Endoscopic Score for Crohn’s Disease; HBI - Harvey Bradshaw Index; CDAI - Crohn’s Disease Activity Index.

^*^Spearman rank correlation coefficient.

### Adropin as a predictor for positive IBD status

A multivariable logistic regression analysis showed that serum adropin was a significant predictor of positive IBD status when enumerated along with baseline characteristics (OR 0.455, 95% CI 0.251–0.823, *P* = 0.009) (Table 5).

**Table 5:** Multivariable logistic regression analysis of independent predictors for positive IBD status.

Variable	OR	95% CI	*P*
Age (years)	1.014	0.975–1.053	0.484
BMI (kg/m^2^)	0.863	0.694–1.073	0.186
Waist circumference (cm)	1.018	0.958–1.082	0.552
Gender	0.853	0.345–2.011	0.731
Adropin (ng/mL)	0.455	0.251–0.823	0.009

Abbreviations: 95% CI- 95% confidence interval; BMI- body mass index; OR- multivariable adjusted odds ratio.

## Discussion

Our study showed that patients with inflammatory bowel disease had significantly lower serum adropin levels compared to the control group, while there was no significant difference between UC and CD adropin levels. Furthermore, adropin had a significant negative correlation with several IBD severity scores and with multivariable logistic regression was a significant predictor of positive IBD status. However, multivariable logistic regression indicates an association but doesn’t prove causality. To the best of our knowledge, this is the first study that evaluates adropin in patients with IBD.

Adropin is a peptide mostly associated with energy homeostasis and vascular protection but through its network of pathways and interactions it could also be linked with inflammation. Recently several studies reported a correlation between serum adropin and different disorders such as diabetes mellitus, atherosclerosis, polycystic ovary syndrome and obstructive sleep apnea^19–21,32^. Furthermore, all of these disorders are associated with a low-grade chronic inflammation, downstream of pro-inflammatory cytokines and endothelial dysfunction^32–34^. Given that endothelial dysfunction is involved in IBD pathogenesis and since it is well documented that adropin has a role in neovascularization and vascular protection through regulation of VEGF and eNOS pathways^11^, these findings could be pointing that one of the missing links between inflammation and endothelial dysfunction could be adropin. It is possible that inflammation is the one that downregulates adropin through the fallout of pro inflammatory cytokines, most prominently TNF-α, which consequently leads to endothelial dysfunction. As mentioned before adropin regulates production of nitric oxide (NO) and it is well established that endothelial dysfunction in IBD patients is associated with the decreased production of NO, whereas studies showed that administration of anti TNF-α therapy in IBD leads to significant improvement of endothelial dysfunction^35,36^. This could also explain why the risk of coronary heart disease and heart failure is higher in IBD patients^37,38^, while the prevalence of traditional cardiovascular risk factors, such as high BMI, diabetes, hyperlipidemia, obesity, hypertension and lack of physical activity is relatively lower in IBD patients than in the general population^39^. Another possible mechanism through which adropin could be associated with IBD pathophysiology is its antioxidative effect. A recent study conducted on mice with induced nonalcoholic steatohepatosis showed that knockout of adropin significantly exacerbated steatosis, inflammation and fibrosis while on the other hand intraperitoneal administration of adropin led to reduced expression of inflammation genes and upregulated nuclear factor erythroid 2-related factor 2 (Nrf2)^40^, one of the most prominent regulators of cellular resistance to oxidative stress^41^. It is possible that adropin in a similar way of action through Nrf2 alleviates oxidative stress which is, proven by several studies, closely connected with inflammation in IBD^42,43^. Nevertheless, additional studies are needed to clarify the role of adropin in IBD patients and its part in this complex cascade of pathophysiological pathways.

Contrary to our expectations, we didn’t find a significant correlation between adropin and hsCRP in IBD patients. It is acknowledged that even so CRP is insufficient to substitute endoscopic or radiographic findings in IBD, it’s well used for evaluation of the disease severity^44^. All of the previously mentioned studies on low-grade inflammatory conditions found the presumed association^19–21,30^. This result could be circumstantial due to inclusion of only those patients who had a stable IBD activity in the past 3 months.

Another interesting finding of our study was the negative correlation between adropin and FC. Several studies showed that FC correlates strongly with the laboratory and diagnostic signs of disease activity^45,46^. Adropin correlation with FC implies that adropin could be associated with IBD activity and severity, especially when enumerated with our finding of adropin significant association with UC and CD severity scores, which are the clinically among most important instruments for evaluation of the disease activity. However, it is also possible that these results are due to adropin down regulation through previously mentioned inflammation mechanism. Higher disease activity amplifies FC levels but also reactively up regulates proinflammatory cytokines such as TNF-α and possibly subsequently reduces adropin levels.

Additional significant finding of our study is the negative correlation of serum adropin levels with fasting glucose levels in the whole study population. That is in line with the established fact that adropin is a regulator of glucose and energy homeostasis^47^. A recent study on mice showed that intraperitoneal administration of adropin results in a significant reduction of blood glucose, serum insulin levels and increase of serum adropin levels^48^. Another animal study showed that there is an inverse relationship between adropin levels and dysregulation of glucose metabolism^22^. However, a study conducted on human participants reported an increase of plasma adropin levels with fructose intake which was most prominent in subjects who exhibited hypertriglyceridemia^49^. In our recent study on patients with obstructive sleep apnea we also found a negative correlation between adropin and fasting glucose levels and furthermore a negative correlation between adropin and HOMA-IR and HbA1c. We discussed this through the possibility of adropin interactions with eNOS and therefor increased systemic bioavailability of NO. Consequently, decreased adropin would lead to lower insulin sensitivity and increased inflammation burden as showed by adropin correlation with HOMA-IR, TNF-α and IL-6 in that study^32^. Furthermore, a recent study showed that adropin treatment reduced expression of gluconeogenic regulatory enzymes in the liver which subsequently inhibited hepatic glucose production while improving hepatic insulin sensitivity^50^. This is in alignment with the results of an animal study which proved that adropin levels are higher in fed mice and lower in fasting ones^48^. It is possible that adropin is down regulated by fasting conditions so gluconeogenesis wouldn’t be inhibited and peripheral fatty acid would be used to support hepatic glucose production, while blood glucose would be spared to support the central nervous system. On the contrary, during re‐feeding adropin levels increase and glucose utilization is activated.

The limitation of our study was its cross-sectional design which disables establishment of causal relationship. Moreover, it had single center patient inclusion with a relatively small sample size. Additionally, due to technical reasons we were not able to measure FC in the whole control group.

In conclusion, this is the first study that reported decreased serum adropin levels in patients with IBD and demonstrated a negative correlation between adropin levels and IBD severity scores. Altogether our study implies that adropin could be involved in the complex pathophysiology of IBD and even potentially serve as a novel predictor of the disease activity. However, future larger scale studies are necessary to evaluate significance of these findings.
